# Discrepancy Between Biological Activity and Functional Fracture Healing Following Vitamin K2 Supplementation in an Ovariectomized Rat Model of Osteoporosis

**DOI:** 10.3390/jcm15124510

**Published:** 2026-06-10

**Authors:** Alexandru Jecan, Răzvan Marian Melinte, Gheorghe Tomoaia, Luciana-Mădălina Gherman, Vasile Rus, Raluca Maria Pop, Cătălin Popa, Diana Jecan-Toader, Dragoș Apostu, Marian Andrei Melinte, Daniel Oltean-Dan

**Affiliations:** 1 Department of Orthopedics and Traumatology, University of Medicine and Pharmacy “Iuliu Hațieganu”, 400132 Cluj-Napoca, Romania; jecan.alexandru@elearn.umfcluj.ro (A.J.); contact@drapostu.ro (D.A.); olteandandaniel@yahoo.com (D.O.-D.); 2 Orthopedics and Traumatology Clinic “Alexandru Radulescu”, Emergency County Hospital, 400347 Cluj Napoca, Romania; 3 Experimental Centre, University of Medicine and Pharmacy “Iuliu Hațieganu”, 400349 Cluj-Napoca, Romania; luciana.gherman@umfcluj.ro; 4 Department of Histology, Faculty of Veterinary Medicine, University of Agricultural Sciences and Veterinary Medicine, 400372 Cluj-Napoca, Romania; vasile.rus@usamvcluj.ro; 5 Department of Pharmacology, Toxicology and Clinical Pharmacology, University of Medicine and Pharmacy “Iuliu Hațieganu”, 400012 Cluj-Napoca, Romania; raluca_parlog@yahoo.com; 6 Department of Materials Science and Engineering, Technical University of Cluj-Napoca, 400641 Cluj-Napoca, Romania; 7 EUt+ Institute of Nanomaterials & Nanotechnologies EUTINN, European University of Technology, European Union; 8 Department 2, Faculty of Nursing and Health Sciences, University of Medicine and Pharmacy “Iuliu Hațieganu”, 400132 Cluj-Napoca, Romania; 9 2nd Pediatric Clinic, Emergency Clinical Hospital for Children, 400124 Cluj-Napoca, Romania; 10 Faculty of Medicine, University of Medicine and Pharmacy “George Emil Palade”, 540139 Târgu Mureș, Romania; marianmelinte01@gmail.com

**Keywords:** osteoporosis, fracture, vitamin K2, MK-4, MK-7, ovariectomized rat model, bone strength, bone quality, osteocalcin

## Abstract

**Background**: Vitamin K2 (menaquinone) has been studied as a molecule with important effects on bone metabolism and has been proposed as a potential adjuvant in fracture healing, particularly under osteoporotic conditions. However, its functional impact on osteoporotic fracture healing remains largely undefined. The aim of this study was to evaluate the effects of vitamin K2 supplementation, in the form of menaquinone-4 (MK-4) and menaquinone-7 (MK-7), on fracture healing in an ovariectomized rat model of osteoporosis. **Methods**: Forty Wistar rats were included in this study and allocated to four equal groups: Sham control, ovariectomized control, MK-4, and MK-7. Osteoporosis was induced by bilateral ovariectomy, and 12 weeks after ovariectomy, a femoral fracture was produced and fixed by intramedullary nailing. Starting on postoperative day 2, the MK-4 group received 5 mg/kg/day of MK-4, while the MK-7 group received MK-7 at a dose of 0.05 mg/kg/day. Fracture healing was assessed primarily by biomechanical testing using a three-point bending test and was further analyzed by histological and biochemical parameters, including CTXI, PINP, ucOC, BALP, and ALT. **Results**: Vitamin K2 supplementation did not improve functional fracture healing. In both treatment groups, fractures showed nonunion-like mechanical behavior, precluding meaningful quantitative biomechanical comparison. Although histological and biochemical findings, particularly in the MK-4 group, showed some degree of biological activity, these changes did not translate into mechanically competent bone union. Both treatment groups showed a tendency toward impaired healing, with progression toward nonunion-like behavior under the present experimental conditions. No significant hepatic toxicity was observed. **Conclusions**: In this ovariectomized rat femoral fracture model, vitamin K2 supplementation with either MK-4 or MK-7 did not enhance functional fracture healing despite evidence of biological activity of the treatment. These findings suggest a discrepancy between molecular or histological effects and biomechanical outcomes, indicating that, under the conditions tested, vitamin K2 is insufficient to overcome impaired healing in osteoporotic bone and may adversely influence fracture repair under these experimental conditions, although the mechanism remains uncertain.

## 1. Introduction

Osteoporosis and postmenopausal estrogen deficiency are well known to impair bone strength and fracture repair. In ovariectomized (OVX) rodent models, fracture healing is often delayed. These models show formation of larger but mechanically weaker calluses and exhibit delayed remodeling [1]. Vitamin K2 (menaquinones) is essential for bone health because it promotes ɣ-carboxylation of osteocalcin. In addition, menaquinones play an important role in bone matrix production and collagen synthesis [2]. Studies show that vitamin K2 supplementation, including menaquinone-4 (MK-4) and menaquinone-7 (MK-7), improves bone quality. In OVX mice, MK-4 treatment significantly increased alkaline phosphatase activity and bone volume, improved bone mineral density (BMD), and reduced undercarboxylated osteocalcin (ucOC) [3]. Likewise, MK-7 appears to enhance bone quality primarily rather than BMD. Long-term MK-7 supplementation in OVX rats prevented trabecular bone loss and significantly improved bone strength [2,4]. In humans, supplementation with 45 mg/day of MK-4 or 180 μg/day of MK-7 has also been reported to maintain bone strength despite minimal changes in BMD [5,6].

Despite this evidence supporting improved bone status, the effects of MK-4 and MK-7 on osteoporotic fracture healing remain less well documented. In non-OVX rats, high-dose MK-4 promoted lamellar bone formation within the callus and was associated with downregulation of bone turnover, favoring more stable repair [7]. Similarly, in a rat tibial fracture model, vitamin K2 significantly increased callus strength and radiographic healing scores [8]. Although these findings suggest a beneficial effect of vitamin K2 on fracture healing, it remains unclear how MK-4 and MK-7 influence fracture repair under osteoporotic conditions.

In addition to structural and histological evaluation, assessment of biochemical markers may provide further insight into the biological response to treatment during fracture healing. Biomechanical testing and histology mainly reflect the structural and functional consequences of remodeling, whereas bone turnover markers (BTMs) can capture more dynamic changes in bone metabolism. The European Society for Clinical and Economic Aspects of Osteoporosis (ESCEO) and the International Osteoporosis Foundation (IOF) recommend utilizing procollagen type I N-terminal propeptide (PINP), bone alkaline phosphatase (BALP), and C-terminal telopeptide of type I collagen (CTXI) as reference BTMs for osteoporosis [9]. Moreover, ucOC represents an important marker for Vitamin K activity in bone [10]. In parallel, alanine aminotransferase (ALT) was assessed, together with histological examination, to provide information regarding the hepatic safety profile of the intervention [11].

To address this gap, we conducted an experimental study of mid-diaphyseal femoral fracture healing in OVX rats treated with MK-4 and MK-7. The aim of the study was to investigate the effects of vitamin K2 on osteoporotic fracture healing and to evaluate its hepatic safety profile.

## 2. Material and Methods

### 2.1. Ethical Statement

All animal experiments were performed in accordance with internationally recognized ethical standards and complied with European Union directive 2010/63/EU on the protection of animals used for scientific purposes. Ethical approval was granted by the Institutional Ethics Committee of the “Iuliu Hatieganu” University of Medicine and Pharmacy, Cluj-Napoca (approval no. 13/02 February 2024), and by the National Sanitary Veterinary and Food Safety Authority (ANSVSA) (approval no. 397/28 February 2024).

The study included forty female Wistar albino rats, 7 weeks of age, with body weights ranging from 145 to 185 g. Animals were housed in standard polycarbonate cages under controlled environmental conditions: temperature 22 ± 2 °C, relative humidity of 55 ± 5%, and a 12 h/12 h light-dark cycle. Food and water were available ad libitum. Throughout the study, animal handling was performed using tunnels and other refined, non-aversive techniques to reduce stress.

All operative procedures and euthanasia were conducted under veterinary oversight and in accordance with European animal welfare regulations, with every effort made to reduce suffering.

### 2.2. Experimental Design

After a one-week acclimatization period, the rats were randomly assigned using a computer-generated randomization sequence to four equal groups (*n* = 10/group):

Group 1 (Sham control): sham ovariectomy + femoral fracture, treated with corn oil.

Group 2 (OVX+FR control): bilateral ovariectomy + femoral fracture, treated with corn oil.

Group 3 (OVX+FR+MK-4): bilateral ovariectomy + femoral fracture, treated with MK-4 at 5 mg/kg/day.

Group 4 (OVX+FR+MK-7): bilateral ovariectomy + femoral fracture, treated with MK-7 at 0.05 mg/kg/day.

The primary outcome of the study was functional fracture healing, assessed by biomechanical behavior during three-point bending testing, including the ability to obtain an interpretable load-to-failure curve consistent with mechanically competent union. Biochemical markers, histological assessment, and hepatic safety evaluation were considered secondary and exploratory outcomes.

Sample size rationale

This study was designed as an exploratory animal study. Because no directly comparable full-length study evaluating MK-4 and MK-7 supplementation in an ovariectomized osteoporotic fracture-healing model was available to provide a reliable expected effect size for biomechanical healing, a formal a priori sample size calculation was not performed. The initial group size of 10 animals per group was selected based on feasibility, a comparable experimental fracture-healing model, and ethical principles aimed at reducing animal use while allowing preliminary intergroup comparison. After attrition and standardization, eight animals per group were included in the final analyses.

#### Surgical Procedure

All surgical procedures were performed under aseptic conditions by a blinded investigator using sterile instruments and disposable drapes. Animals were weighed preoperatively for dose adjustment, and general anesthesia was induced by intraperitoneal ketamine (75 mg/kg) and medetomidine (0.5 mg/kg). After adequate anesthesia was achieved, rats were positioned in dorsal recumbency, and the ventral abdomen was shaved and disinfected with 10% povidone-iodine solution. A midline laparotomy of approximately 5 cm was performed, with electrocautery used for hemostasis. After gentle lateral retraction of the bowel loops, the ovaries were exposed. In OVX animals, both ovaries and the adjacent broad ligament were isolated and bilaterally transected by electrocautery, followed by complete excision of the ovaries. In sham-operated animals, the ovaries were exposed in the same manner but were not removed.

The abdominal cavity was irrigated with prewarmed sterile saline (0.9% NaCl) before layered closure. The abdominal wall was closed with continuous locking 2/0 Vicryl (polyglactin 910) sutures, and the skin was closed with simple interrupted 2/0 polypropylene sutures. The wound was disinfected with povidone-iodine and sprayed with topical oxytetracycline. After surgery, animals were kept in a temperature-controlled recovery area and observed until full recovery of the righting reflex and spontaneous locomotor activity.

Twelve weeks after ovariectomy, to allow for the development of osteoporotic changes, all animals underwent the same intended standardized femoral osteotomy and fixation protocol under general anesthesia. Using a lateral approach, the osteotomy was performed in the middle third of the femur. A transverse mid-diaphyseal osteotomy was performed with a saw, and fracture stabilization was achieved by retrograde insertion of an intramedullary titanium implant through the intercondylar femoral notch into the medullary canal. Although the surgical technique and fixation were standardized, final fracture morphology was assessed descriptively rather than by a predefined radiographic or histological fracture complexity score.

The intramedullary implants consisted of titanium alloy rods (Grade 5 titanium/aluminum/vanadium alloy, Ti90/A1 6/V 4; Goodfellow GmbH, Alstertwiete 3, D-20099 Hamburg, Germany). The original 1000 mm × 1 mm rods were cut into 20 mm × 1 mm segments, which were subsequently sterilized by autoclaving before implantation. After fixation, the surgical wound was closed in layers and disinfected with povidone-iodine solution, and tetracycline was sprayed locally. Postoperatively, the animals were kept in a temperature-controlled recovery area and were monitored closely during the recovery period.

### 2.3. Treatment Administration

Treatment was initiated on postoperative day 2 of the second surgery and continued for 6 weeks. This timing was selected to evaluate the effect of vitamin K2 on the healing of an already established osteoporotic fracture. No pre-fracture loading phase was used, because supplementation during the 12-week post-ovariectomy period could have modified the development or severity of estrogen-deficiency-induced osteoporosis before fracture creation and thereby confounded the assessment of post-fracture healing. Animals in the treatment groups received vitamin K2 solutions via oral gavage once daily. The MK-4 group was treated with menaquinone-4 at a dose of 5 mg/kg/day, while the MK-7 group received menaquinone-7 at a dose of 0.05 mg/kg/day. The dose rationale was based on adjacent preclinical and translational evidence because, at the time of protocol design, no directly comparable study evaluating MK-4 and MK-7 supplementation in an ovariectomized osteoporotic fracture-healing model was identified. Therefore, the selected doses were derived from studies assessing vitamin K2 effects in ovariectomy-induced bone loss, MK-4 skeletal distribution, and MK-7 biological activity in experimental models. For MK-4, the dose of 5 mg/kg/day was chosen based on rodent data showing biological activity and bone distribution of orally administered MK-4. Sano et al. reported that repeated oral administration of MK-4 at 4 mg/kg/day in rats resulted in accumulation in bone marrow and cancellous bone, including in ovariectomized animals, with bone concentrations comparable to pharmacologically effective concentrations described in bone formation studies [12]. Higher MK-4 doses have also been used in ovariectomized rodent models, including 20–40 mg/kg every other day, supporting that the present dose was within a biologically relevant but comparatively moderate preclinical range. For MK-7, the dose of 0.05 mg/kg/day was selected because low-dose MK-7 regimens of 0.03–0.05 mg/kg/day have been reported to exert biological effects in mice after metabolic-rate adjustments and have been considered compatible with effective human supplemental doses of longer-chain menaquinones. Because MK-4 and MK-7 differ substantially in pharmacokinetics, bioavailability, circulating half-life, tissue distribution, and commonly used experimental dose ranges, the doses were not intended to be directly mass-equivalent or osteogenically equivalent. The present study should therefore be interpreted as an exploratory comparison of two vitamin K2 isoforms administered at literature-supported biologically relevant doses, rather than as a direct dose-equivalence study [3,12,13,14]. Control animals received an equivalent volume of corn oil without active compound. Administration volumes were adjusted according to body weight, which was measured each week. All treatments were administered by a blinded investigator.

For both MK-7 and MK-4, stock solutions were prepared in corn oil, after which working solutions were obtained by dilution with corn oil preheated to 40 °C to improve solubilization and homogeneity. To preserve compound stability, fresh working solutions were prepared on a weekly basis throughout the treatment period. Because the animals’ body weight changed over time, rats were weighed weekly and the gavage volume was adjusted to maintain the planned dose per kilogram. Prior to each administration, the solutions were gently reheated and mixed to restore homogeneity and redissolve any material that had precipitated during refrigerated storage.

### 2.4. Experimental Groups, Attrition and Sample Collection

At the end of the 18-week experimental period, 36 of the 40 initially enrolled rats survived. During the first phase of the study, including the intraoperative and immediate perioperative period of the first surgical procedure, three deaths occurred: two in the OVX+FR+MK-7 group and one in the sham control group. A further death occurred during the second surgical intervention in the OVX+FR control group. Thus, the surviving animals were distributed as follows: Sham control (*n* = 9), OVX+FR control (*n* = 9), OVX+FR+MK-4 (*n* = 10), OVX+FR+MK-7 (*n* = 8). For standardization of sample collection and analysis, an equal number of animals was included from each group. In groups with more than 8 surviving rats, each animal was assigned a number from 1 to *n* and those excluded from the analysis were selected using a computer-generated random number. These exclusions were performed for group-size standardization only and were not based on biochemical, histological, or biomechanical outcomes.

At the end of the 6-week post-fracture treatment period, blood samples were collected from the caudal vein under light sedation to reduce handling-related stress. Samples were drawn into EDTA-containing tubes and centrifuged within 20 min of collection at 2500 rpm (approximately 650× *g*) for 5 min to obtain plasma. The separated plasma was aliquoted and stored at −80 °C until further biochemical determinations. Due to hemolysis, plasma samples from four Sham control animals could not be used for biochemical determinations. Therefore, biochemical analysis included *n* = 4 animals in the Sham control group and *n* = 8 animals in each OVX fracture study group, whereas mechanical testing and histological endpoint allocation remained at *n* = 4 per group. Following blood sampling, the animals were euthanized by anesthetic overdose, ensuring a rapid and humane death in accordance with ethical guidelines. Immediately thereafter, the liver and left femur were harvested. The femurs were dissected free of surrounding soft tissue and fixed in 10% neutral buffered formalin for later histological and biomechanical analyses, while liver specimens were fixed in 10% neutral buffered formalin for histopathological evaluation of systemic toxicity. Femurs were randomly assigned using a computer-generated random number into a histological analysis group (*n* = 4) or mechanical testing group (*n* = 4).

### 2.5. Bone Turnover Markers

All samples were analyzed by a blinded researcher. Plasma concentrations of bone turnover markers were assessed using ELISA kits supplied by ELK Biotechnology (Wuhan, China), in accordance with the manufacturer’s instructions. CTXI was measured by competitive inhibition ELISA (ELK2221; sensitivity 49.6 pg/mL; range 156.25–10,000 pg/mL), whereas BALP (ELK5682; sensitivity 0.3 ng/L; range 0.79–50 ng/mL), PINP (ELK7661; sensitivity 1.18 ng/mL; range 3.13–200 ng/mL), and ucOC (ELK9272; sensitivity 0.058 ng/mL; range 0.16–10 ng/mL) were measured using sandwich ELISA assays. Absorbance was recorded with an 800 TS ELISA microplate reader, and washing steps were performed using a BioTek 50 TS plate washer (both Agilent Technologies Inc., Santa Clara, CA, USA). ALT (REF 11533) was analyzed spectrophotometrically on an Applied Biosystems A15 automated analyzer (Costa Brava, Barcelona, Spain) to assess potential liver toxicity.

### 2.6. Mechanical Testing

To assess bone mechanical behavior, three-point bending tests were performed using a Zwick/Roell Z005 machine (Zwick/Roell, Ulm, Germany), with data acquisition carried out using TestXpert II software (Zwick/Roell, Ulm, Germany). The femurs were supported at the metaphyseal regions, and a progressively increasing load was applied in the anteroposterior plane at the level of the fracture, located in the mid-diaphysis area, at a constant displacement rate of 1 mm/min until failure. Maximum load-to-failure was recorded for each specimen.

### 2.7. Histology

All samples were assessed by a blinded researcher. Histological evaluation was performed as a qualitative descriptive assessment of callus morphology, tissue composition, fragment alignment, and liver histology. Quantitative histomorphometry, including BV/TV, trabecular thickness, cartilage area fraction, and fibrous tissue area fraction, was not part of the predefined protocol. Photomicrographs were obtained from systematically selected representative fields. Following euthanasia, liver and femur specimens were collected for histopathological evaluation. To facilitate penetration of the fixative into the medullary canal, the femoral epiphyses were resected before fixation and the intramedullary nail extracted. Liver tissue fragments of approximately 5 mm thickness and femoral diaphyseal specimens were fixed in 10% neutral buffered formalin at room temperature for 5 days. After fixation, bone samples underwent decalcification in 5% trichloroacetic acid for 30 days. Subsequently, all tissues were processed routinely by dehydration in graded ethanol, clearing in n-butanol, and paraffin embedding. Serial 5 μm sections were prepared using a rotary microtome (Leica RM 2125; Leica Biosystems, Nussloch GmbH, Nussloch, Germany) and mounted on glass slides.

Histological staining was performed using Goldner’s trichrome. Slide evaluation was carried out with an Olympus BX41 light microscope (Olympus, Tokyo, Japan), and digital photomicrographs were obtained using Olympus cellSens Entry 3.1 software.

### 2.8. Statistical Analysis

Statistical analyses were performed using SPSS (Version 26, release 26.0.0.0 64 bit edition) (IBM Corp, Armonk, NY, USA). Data distribution was assessed with the Shapiro–Wilk test and by visual inspection of histograms and Q-Q plots. Homogeneity of variances was examined using Levene’s test. Continuous variables with normal distribution are presented as mean ± SD, whereas non-normally distributed variables are presented as median (IQR). Intergroup comparisons were performed using one-way ANOVA when normality and homogeneity of variances assumptions were met, with Tukey’s post hoc test for pairwise comparisons. When variances were unequal, Welch’s ANOVA with Games–Howell post hoc correction was applied. Variables with a non-normal distribution were analyzed using the Kruskal–Wallis test followed by Dunn–Bonferroni post hoc comparisons. When only two groups with non-normally distributed data were compared, the Mann–Whitney U test was used. Statistical significance was set at *p* < 0.05.

## 3. Results

### 3.1. Biochemical Endpoints

Data distribution was normal for C-terminal telopeptide of type I collagen (CTXI), procollagen type I N-terminal propeptide (PINP), undercarboxylated osteocalcin (ucOC), and alanine aminotransferase (ALT) and non-normally distributed for bone alkaline phosphatase (BALP).

Descriptive values for ALT, CTXI, ucOC, BALP and PINP across the four study groups are presented in Table 1 and Supplementary Figure S1. Individual animal-level biochemical values are provided in Supplementary Table S1.

No significant differences in ALT levels were observed among the groups (Welch’s F = 1.741, *p* = 0.185). Likewise, BALP levels did not differ significantly between groups (Kruskal–Wallis, H(3) = 2.171, *p* = 0.538). In contrast, CTXI (Welch’s F = 19.859, *p* < 0.001), ucOC (Welch’s F = 12.292, *p* = 0.001) and PINP (Welch’s F = 10.152, *p* = 0.002) differed significantly among groups (Table 2).

Global intergroup comparisons showed significant differences for CTXI, ucOC, and PINP, whereas ALT and BALP did not differ significantly among groups (Table 2). Games–Howell post hoc comparisons are indicated by superscript letters in Table 1. CTXI was significantly higher in the sham-operated group than in all OVX fracture groups, with no significant differences among the three OVX fracture groups. For ucOC, significant differences were observed between the sham-operated group and the OVX+FR control group, and between the OVX+FR control group and the OVX+FR+MK-4 group. For PINP, the OVX+FR+MK-4 group showed significantly higher values than both the sham-operated and OVX+FR control groups. No other pairwise comparisons reached statistical significance.

### 3.2. Histology

#### 3.2.1. Bone Histology

**Figure 1:**
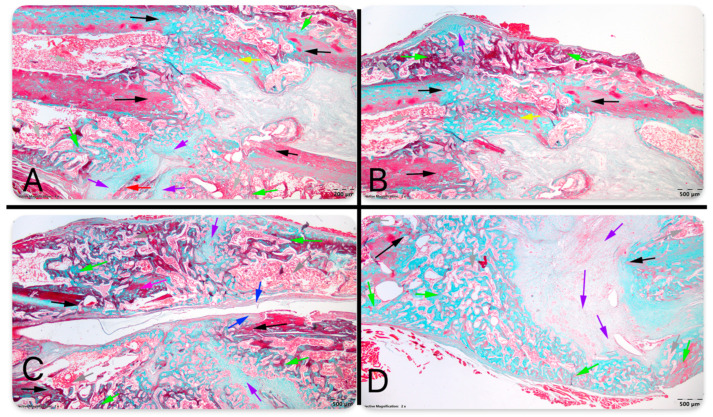
Histological features of fracture repair in OVX+FR control. Goldner’s trichrome stain. Pannels (**A**–**D**): black arrows: fracture diaphyseal ends; green arrows: subperiosteal callus; gray arrows: hematopoietic marrow; purple arrows: periosteal fibrocartilaginous callus; yellow arrow: medullary-displaced fragment. (**A**): red arrow: subperiosteally displaced bone fragment; (**C**): blue arrow: fibrous tissue proliferated in the medullary cavity around the inserted rod; pink arrow: bone fragment located along the fracture line. Scale bar is shown in the lower right corner of each panel. A consolidated arrow-color legend is provided in Supplementary Table S2.

**Figure 2:**
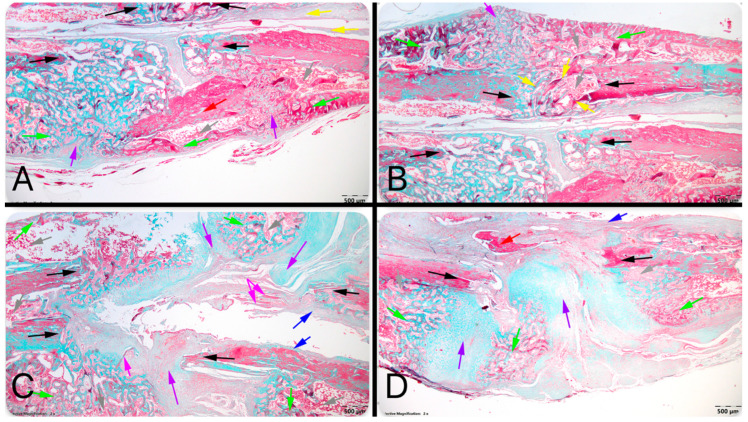
Histological features of fracture repair in OVX+FR+MK-4. Goldner’s trichrome stain. Black arrows: fracture diaphyseal ends; green arrows: subperiosteal callus; gray arrows: hematopoietic marrow; purple arrows: periosteal fibrocartilaginous callus. (**A**): yellow arrow: fibrous tissue proliferating around the inserted rod; red arrow: subperiosteally displaced fragment; (**B**): yellow arrow: osseous callus proliferating between the fractured diaphyseal ends. (**C**): pink arrow: bone fragment located along the fracture line; blue arrow: fibrous tissue proliferating within the medullary cavity around the inserted rod. (**D**): red arrow: osseous displaced fragment in the medullary cavity; blue arrow: fibrous tissue proliferating around the inserted rod. Scale bar is shown in the lower right corner of each panel. A consolidated arrow-color legend is provided in Supplementary Table S2.

**Figure 3:**
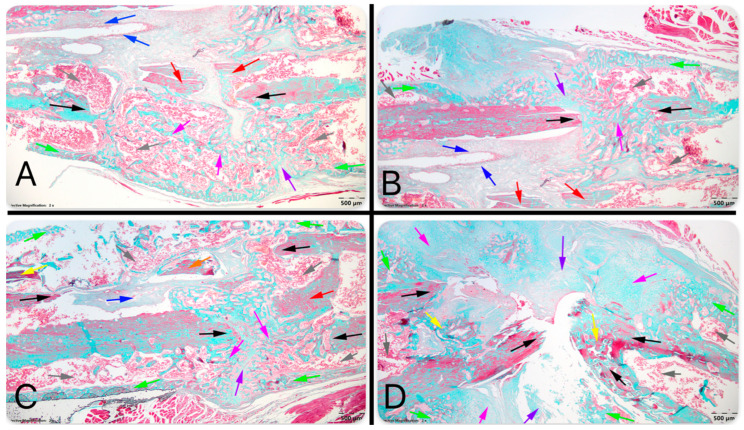
Histological features of fracture repair in OVX+FR+MK-7. Goldner’s trichrome stain. Black arrows: fracture diaphyseal ends; green arrows: subperiosteal osseous callus; gray arrows: hematopoietic marrow; purple arrows: periosteal fibrocartilaginous callus; blue arrows: fibrous tissue proliferating around the rod; red arrow: osseous displaced fragment in the medullary cavity. (**A**): pink arrow: osseous callus proliferating from the medullary cavity; (**B**): pink arrow: osseous callus proliferating from the medullary cavity; (**C**): pink arrow: osseous callus proliferating from the medullary cavity; orange arrow: osseous fragment remaining along fracture line; (**D**): pink arrow: fibrocartilaginous callus proliferating from the periosteum medial to the fracture line. Scale bar is shown in the lower right corner of each panel. A consolidated arrow-color legend is provided in Supplementary Table S2.

Bone histology was evaluated descriptively. No quantitative histomorphometry measurements were performed. Therefore, the following findings should be interpreted as qualitative morphological observations. Microscopic examination of bone specimens showed variable fracture configuration, including transverse and oblique comminuted fractures, with displaced fragments located either subperiosteally or within the medullary cavity, as well as cases with partial longitudinal alignment of fragments and fractures with non-opposed and/or misaligned ends. These fracture pattern differences were documented descriptively; no quantitative comminution or alignment score was available for intergroup comparison. A consolidated arrow-color legend for Figure 1, Figure 2 and Figure 3 is provided in Supplementary Table S2 to improve readability of the histological annotations. Across all analyzed groups, the fracture callus was composed predominantly of trabecular bone associated with hematopoietic marrow, together with variable amounts of fibrocartilaginous tissue. A consistent pattern across OVX+FR+Control, OVX+FR+MK-4 and OVX+FR+MK-7 was that newly formed osseous tissue appeared to arise predominantly from the endosteal side, whereas fibrocartilaginous tissue was mainly distributed periosteally and along the fracture line.

In the OVX+FR control group, the histological appearance was heterogeneous and depended on fragment position and alignment. When bone fragments were displaced subperiosteally, they were surrounded by fibrocartilaginous callus, while the fractured diaphyseal ends were encased externally by trabecular osseous callus of moderate thickness with relatively large marrow-filled areolae (Figure 1A). When fragments were displaced into the medullary cavity, the callus between fracture ends was composed mainly of trabecular bone, with somewhat thicker trabeculae than in the subperiosteal displacement pattern and only limited fibrocartilaginous tissue at the periosteal aspect of the fracture line (Figure 1B). In fractures with aligned fragments, the callus was again mainly osseous, with residual fibrocartilaginous tissue persisting focally along the fracture line (Figure 1C). In non-opposed or misaligned fractures, larger fibrocartilaginous areas were present centrally, especially on the open-angle side, where fibrocartilaginous tissue could span much of the gap thickness, whereas osseous callus remained more peripheral (Figure 1D). Overall, this group showed mixed repair, with variable osseous consolidation and persistent fibrocartilaginous tissue at the fracture site.

In OVX+FR+MK-4, the callus was also predominantly trabecular but histologically appeared more consolidated than in OVX+FR control. In comminuted fractures with subperiosteally displaced fragments, the callus between fracture ends consisted mainly of trabecular bone with relatively thick trabeculae and only limited fibrocartilaginous tissue extending superficially from the periosteal side (Figure 2A). In areas where the distance between fracture fragments was small, osseous tissue clearly predominated, with only small focal cartilaginous components (Figure 2B). In more complex or non-opposed fractures, fibrocartilaginous tissue was still present, sometimes over extended areas, particularly around small unstable fragments or across larger fracture gaps, but the peripheral osseous component remained well developed (Figure 2C,D). Compared with control group, OVX+FR+MK-4 group qualitatively appeared to show a larger callus diameter, a greater surface area occupied by osseous tissue, thicker trabeculae and a higher apparent degree of callus consolidation.

In the OVX+FR+MK-7 group, the callus occupied a broad area in most specimens and was predominantly composed of trabecular bone; however, the trabeculae were generally thinner than those observed in OVX+FR control and OVX+FR+MK-4 groups. Fibrocartilaginous tissue persisted along portions of the fracture line, especially periosteally and in regions with greater interfragmentary distance or fragment displacement (Figure 3A,B). In oblique comminuted fractures with medullary displacement of fragments, osseous tissue occupied most of the section, but thin trabeculae and associated fibrous tissue around the rod and displaced fragments were common (Figure 3C). In non-opposed or misaligned fractures, the callus contained a mixed composition of bone, cartilage, and fibrous tissue, with the fibrous component particularly prominent in the central region of the gap and more extensive on the open angle side (Figure 3D). Compared with the other groups, this group appeared to show the largest overall callus area, together with the thinnest trabeculae and a lower apparent degree of maturation of the osseous component.

Taken together, the histological assessment demonstrated that all groups developed mixed reparative tissue composed of osseous and fibrocartilaginous callus. The OVX+FR+MK-4 group appeared to show the greatest degree of apparent consolidation and the thickest trabeculae, whereas the MK-7 group appeared to show the largest callus area but the thinnest trabeculae. The OVX+FR control appeared to have the smallest callus area and an intermediate pattern of trabecular thickness and consolidation. Persistent fibrocartilaginous and fibrous tissue was observed in all groups, especially in fractures with poor fragment alignment or large interfragmentary gaps.

#### 3.2.2. Liver Histology

**Figure 4:**
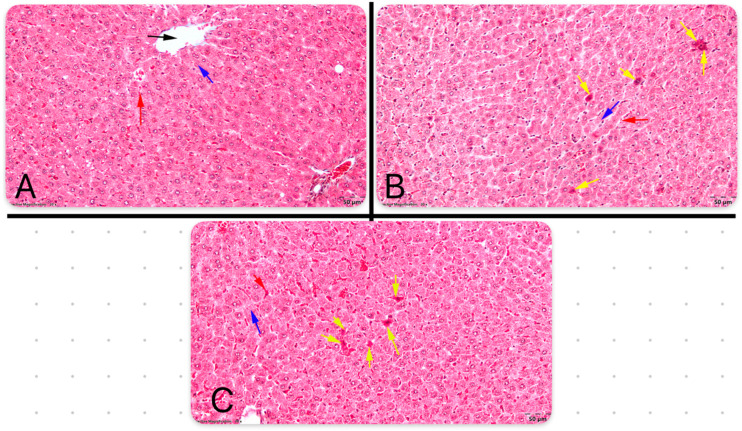
Liver histology. Goldner’s trichrome stain. Pannels (**A**–**C**) of liver histology showing identified and analyzed structures as follows: blue arrows: Remak cords. Red arrows: sinusoidal capillaries; black arrow: centrilobular venule; yellow arrow: apoptotic hepatocytes. Scale bar is shown in the lower right corner of each panel.

In the liver, the OVX+FR control group showed preserved hepatic cytoarchitecture without inflammatory infiltrates or degenerative changes in hepatocytes (Figure 4A). In the OVX+FR+MK-4 group, the only changes were rare apoptotic hepatocytes. In most microscopic fields, no apoptotic cells were observed, and when present, they were usually limited to 1–2 hepatocytes per field, displaying only early apoptotic features, such as mildly eosinophilic cytoplasm and discrete peripheral nuclear hyperchromasia, without advanced nuclear changes (Figure 4B). In the OVX+FR+MK-7 group, apoptotic hepatocytes were even less numerous than in the MK-4 treatment group, and the degree of cytoplasmic eosinophilia and nuclear membrane thickening was less marked (Figure 4C). No inflammatory infiltrates or major structural hepatic lesions were described in any of the analyzed liver groups.

### 3.3. Mechanical Testing

At the six-week post-fracture endpoint, specimens from the OVX+FR+MK-4 and OVX+FR+MK-7 groups showed persistent mobility at the fracture site during manual clinical assessment. During three-point bending testing, these specimens did not generate progressive load–displacement curves compatible with mechanically competent union. Instead, low and fluctuating force values were recorded, with relatively flat curves compared with the sham-operated and OVX+FR control groups. Consequently, load-to-failure values from the treatment groups were considered non-interpretable as measures of united fracture strength, and formal quantitative biomechanical comparison was restricted to the sham-operated and OVX+FR control groups.

**Figure 5:**
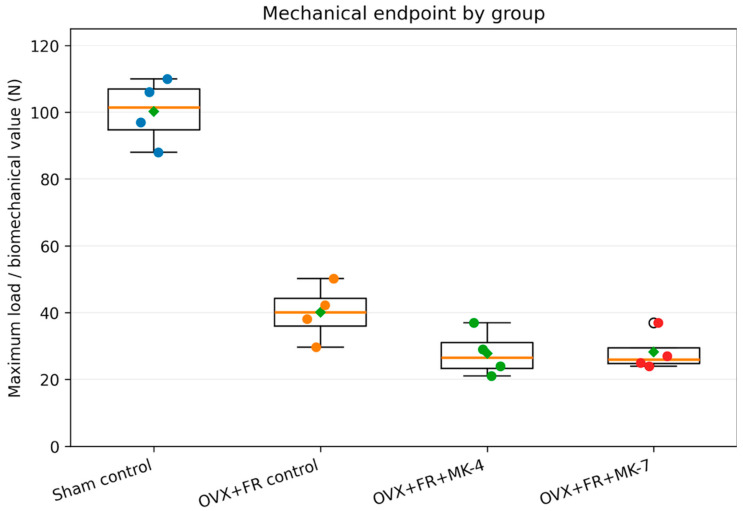
Mechanical endpoint values by experimental group. Individual three-point bending test endpoint values are shown. The Sham-operated group showed higher mechanical competence than OVX+FR controls. Each colored dot represents the tested value of a specimen in the corresponding experimental group. The green diamond in each box represents the mean value of the group. In the treatment groups, specimens showed low, non-interpretable endpoints and nonunion-like mechanical behavior, consistent with failure to achieve mechanically competent union at the six-week endpoint.

In the sham-operated group, the median biomechanical value was 101.500 (IQR 12.250), compared with 40.150 (IQR 8.200) in the OVX+FR control group. Specimens from both groups generated progressive and interpretable load–displacement curves, allowing statistical comparison. However, the significantly lower value observed in the OVX+FR control group indicates reduced mechanical competence rather than normal fracture healing. A significant difference was identified between these two groups (Mann–Whitney U = 16.00, *p* = 0.029), confirming impaired mechanical recovery under estrogen-deficient conditions (Figure 5).

## 4. Discussion

The principal finding of the present study is that, in an ovariectomized rat model of femoral fracture, treatment with MK-4 or MK-7 did not improve functional fracture healing. Although both compounds were associated with a certain degree of biochemical and histological changes suggestive of increased biological activity, more pronounced for MK-4 than for MK-7, these effects did not translate into successful union. Clinically, persistent mobility was observed at the fracture site in the treatment groups, and mechanically, fractures from both OVX+FR+MK-4 and OVX+FR+MK-7 groups showed nonunion-like functional behavior, characterized by low, fluctuating force values and absence of progressive load–displacement curves compatible with mechanically competent union. Consequently, load-to-failure values from these groups were considered non-interpretable as measures of united fracture strength. By contrast, the sham-operated and OVX+FR control groups generated progressive and interpretable load–displacement curves. However, the OVX+FR control group showed markedly lower load-to-failure values than the sham-operated group, indicating impaired mechanical competence rather than normal fracture healing. This supports the validity of the experimental model and confirms the expected detrimental effect of estrogen deficiency on fracture repair.

The union status of the OVX+FR control group should also be interpreted cautiously. Although this group generated interpretable biomechanical data, the load-to-failure value was substantially lower than that of the sham-operated group. Histologically, the OVX+FR control group showed mixed reparative tissue with variable osseous callus and persistent fibrocartilaginous tissue at the fracture site, rather than fully mature cortical restoration. These findings are consistent with impaired repair, incomplete union, or delayed remodeling under estrogen-deficient conditions. Because radiographic or microCT confirmation was not performed, we cannot classify the OVX+FR control group as complete union, delayed union, hypertrophic nonunion, or atrophic nonunion. Nevertheless, this group differed from the MK-4 and MK-7 groups by generating progressive load–displacement curves compatible with mechanically testable repair tissue, and no clinical movement at the fracture site was observed.

Fracture pattern variability should also be considered when interpreting the results. Although the same intended osteotomy and intramedullary fixation protocol was used in all groups, histological evaluation showed variability in the final fracture morphology, including comminution, displacement, malalignment, and differences in interfragmentary distance. Because fracture complexity was not prospectively scored radiographically or histologically, systematic differences between groups cannot be fully excluded. This variability may have contributed to differences in callus morphology, mechanical behavior, and the apparent dissociation between histological activity and functional outcomes.

Prior reports suggest beneficial effects of vitamin K2 on bone metabolism and fracture healing. Previous experimental studies have shown that MK-4 can increase bone volume, BALP activity, and bone mineral density in ovariectomized animals, while MK-7 has been associated with preservation of trabecular structure and improvement in bone strength [3,4]. In non-osteoporotic fracture models, vitamin K2 has also been reported to improve callus quality, radiographic healing, and mechanical strength [7,8]. However, the present study differs from those settings in several important respects, including the use of an ovariectomy-induced osteoporotic model, femoral osteotomy design, fixation method, and focus on functional union as the main endpoint. Moreover, to our knowledge, there are no published full-length studies investigating the effects of vitamin K2 on fracture healing in an osteoporotic animal model. The only comparable study we identified was a 1992 study by Sokol’nikov et al., available to us only as an abstract, which evaluated 1 α (OH)D3, 24R, 25(OH)2D3 and vitamin K of unspecified type in a tibial fracture model in ovariectomized rats [15]. Therefore, the beneficial metabolic effects described in intact bone or less demanding fracture models may not necessarily be sufficient to achieve successful healing under osteoporotic conditions.

An important aspect of the present results is the dissociation between histological appearance and functional outcome. Histologically, the MK-4 group appeared to show the greatest apparent callus consolidation, with thicker trabeculae and a larger osseous area than the OVX+FR control group. The MK-7 group appeared to exhibit the broadest callus area but with thinner trabeculae and a lower apparent degree of maturation. Despite these findings, both treated groups failed to achieve clinically and mechanically competent union. This suggests that increased callus volume or greater trabecular deposition alone is insufficient to indicate effective fracture healing and may instead correspond to a hypertrophic callus pattern in the setting of a nonunion [16,17]. Accordingly, the larger callus observed in the treated groups may reflect persistent reparative activity or delayed remodeling rather than successful consolidation. Therefore, the present findings support the interpretation that MK-4 and MK-7 may have modified the morphology of the reparative response without promoting stable bridging union.

The mechanical findings further support the dissociation between biological activity and effective fracture healing. The sham-operated group showed clearly superior biomechanical performance compared with the OVX+FR control group, confirming the adverse effect of ovariectomy-induced osteoporosis on fracture repair and supporting the validity of the experimental model. In contrast, fractures from the OVX+FR+MK-4 and OVX+FR+MK-7 groups showed persistent fracture-site mobility and failed to generate load–displacement curves compatible with mechanically competent union. These findings indicate that callus formation alone was insufficient to restore functional competence. However, the mechanical behavior of the treatment groups should be interpreted cautiously. Because serial radiographs, pre-testing radiographic assessment, and microCT were not performed, we cannot definitively distinguish delayed union from established radiographic nonunion. Therefore, the findings are best described as nonunion-like functional behavior or failure to achieve mechanically competent union at the six-week endpoint. Although nonunion is a multifactorial process and inadequate stability and osteoporosis are recognized contributing factors, the same surgical technique and fixation method were used in all groups, whereas nonunion was observed only in the treatment groups [18]. Therefore, fixation-related instability or osteoporosis alone is unlikely to be the sole explanation. Possible explanations include delayed remodeling, insufficient mineralized bridging, altered callus composition, treatment-related modification of local repair biology, bone–implant interface effects, or a combination of biological and mechanical factors.

A possible treatment-related interaction with the bone–implant interface should also be considered. Although the same intramedullary titanium implant, surgical approach, and fixation technique were used in all groups, this does not exclude the possibility that MK-4 and MK-7 modified local tissue responses around the implant or fracture gap. In some treated specimens, histological evaluation showed fibrous tissue around the intramedullary rod, raising the possibility that altered interface biology or insufficient osseous integration around the implant contributed to local instability. However, implant loosening, bone–implant contact, fibrous membrane formation, and interface stability were not quantified radiographically, mechanically, or histomorphometrically. Therefore, this explanation remains speculative, but it represents a relevant alternative mechanism for the nonunion-like mechanical behavior observed in the treatment groups.

The CTXI findings should also be interpreted cautiously. In the present study, CTXI values were significantly higher in the sham-operated group than in all OVX fracture groups, while no significant differences were observed among the OVX+FR control, OVX+FR+MK-4, and OVX+FR+MK-7 groups. This pattern was unexpected because ovariectomy is generally associated with increased bone resorption and would typically be expected to increase resorption markers such as CTXI. Several factors may explain this discrepancy, including the single late sampling time point, the dynamic influence of fracture-healing phase on systemic collagen degradation markers, biological variability, systemic rather than local marker assessment, and potential assay-related or pre-analytical limitations. Therefore, CTXI should be considered an exploratory systemic marker in this model and should not be interpreted as definitive evidence of reduced bone resorption in OVX animals or as a robust indicator of vitamin K2 treatment response. In clinical studies, the effect of vitamin K2 on CTXI has also been inconsistent, with systematic reviews reporting modest, null, or heterogeneous effects across populations and study designs. This pattern may suggest that, in the present study, CTXI was driven primarily by the osteoporotic fracture condition rather than vitamin K treatment. Zhang et al., in a recent systematic review and meta-analysis, reported a small but statistically significant reduction in CTXI under vitamin K2 supplementation [19]. However, the effect size was modest and of uncertain clinical relevance. The same review also emphasized that null or inconsistent findings for CTX/NTX are common in the vitamin K2 literature and proposed several possible explanations, including the predominantly formation-oriented biological effects of vitamin K2 and the greater biological and pre-analytical variability of CTXI compared with formation markers such as PINP [19]. In addition, a systematic review by AlHajri et al. found that some included studies reported increased CTXI values from baseline among vitamin K users, further illustrating the heterogeneity of this marker across studies [20]. Taken together, these observations suggest that CTXI may be relatively insensitive or context-dependent for detecting treatment-related effects of vitamin K2, and the findings of the present study are not discordant with existing literature on CTXI and vitamin K2.

The ucOC findings should be interpreted with particular caution. Vitamin K2 promotes osteocalcin carboxylation; therefore, a reduction in circulating ucOC would generally be expected if vitamin K-dependent osteocalcin carboxylation were improved. In the present study, however, ucOC values were lower in the OVX+FR control group than in the sham-operated group, and the only significant difference among OVX fracture groups was observed between OVX+FR control and OVX+FR+MK-4, in a direction that does not support a straightforward improvement in osteocalcin carboxylation. Several factors may explain this unexpected pattern, including the systemic metabolic effects of fracture healing, estrogen-deficiency-related changes in osteocalcin turnover, a single late sampling time point, biological variability, assay-related limitations, and the pre-analytical sensitivity of ucOC. In addition, circulating ucOC may not accurately reflect local vitamin K-dependent osteocalcin carboxylation within the fracture callus. Therefore, the ucOC results should not be interpreted as evidence that MK-4 or MK-7 improved osteocalcin carboxylation under the present experimental conditions [21,22].

Most notably, PINP values were higher in both treatment groups compared to the sham group and the OVX+FR control group. However, statistical significance was reached only in the OVX+FR+MK-4 group compared with the sham and OVX+FR control groups. This finding is consistent with increased osteoblastic activity or matrix synthesis and is in line with reports suggesting that vitamin K2 may stimulate bone formation through PINP-related pathways [19,23]. On the other hand, the systematic review and meta-analysis on vitamin K2 supplementation in middle-aged and elderly populations by Xie et al. found no clear effects on BTMs such as PINP and BALP [24].

Although the literature remains heterogeneous regarding whether vitamin K2 influences bone turnover markers and in what direction, especially in osteoporotic fracture conditions, the present findings suggest that biochemical and histological changes do not necessarily translate into successful fracture healing. In the current study, despite some biochemical and histological features suggestive of a positive biological response, clinical and mechanical union was not achieved. Therefore, these findings should not be interpreted as evidence of improved fracture healing. Rather, the data suggest that MK-4 may have stimulated aspects of matrix production or callus formation without generating functionally competent repair tissue. MK-7 showed a less pronounced biochemical profile, without significant pairwise differences versus the control, which may be consistent with the less mature histological appearance of its callus.

The hepatic safety data were reassuring. ALT values did not differ significantly across groups, and liver histology showed preserved cytoarchitecture in all groups and only rare apoptotic hepatocytes in the treated groups, without inflammatory infiltrates or major structural lesions. These findings indicate that, within the dose range and duration tested, MK-4 and MK-7 were not associated with hepatic toxicity; therefore, the lack of improvement in fracture healing is unlikely to be explained by systemic hepatic intolerance to treatment.

## 5. Limitations

This study has several limitations. First, no meaningful biomechanical dataset could be obtained for the two treatment groups because these specimens showed persistent fracture-site mobility and nonunion-like mechanical behavior at the six-week endpoint. Consequently, treatment comparisons for mechanical performance could not be performed quantitatively. Moreover, serial radiographs, pre-testing radiographic assessment, and microCT were not performed. Therefore, implant loosening, mineralized bridging, callus mineral density, bone–implant contact, and the distinction between delayed union and established radiographic nonunion could not be objectively quantified. In addition, potential treatment-related effects on bone–implant interface biology, including fibrous membrane formation around the intramedullary rod, were not assessed quantitatively. Second, sample sizes were modest, in keeping with ethical considerations regarding animal use. Third, histological evaluation was descriptive rather than quantitative. Standardized histomorphometry, including BV/TV, trabecular thickness, cartilage area fraction and fibrous tissue area fraction within the callus, was not performed. Consequently, qualitative differences in callus morphology and tissue composition should be interpreted cautiously and considered exploratory. Fourth, although the same intended osteotomy and fixation protocol was used in all animals, final fracture patterns showed substantial heterogeneity, including comminution, fragment displacement, malalignment, and differences in interfragmentary distance. Fracture complexity was not prospectively scored using a predefined radiographic or histological classification. Therefore, systematic intergroup differences in fracture morphology cannot be fully excluded and may have contributed to variability in callus formation and mechanical behavior. Fifth, this was an exploratory study and was not based on a formal a priori sample size calculation. Therefore, the final standardized sample size of eight animals per group may have limited statistical power, particularly for biomechanical endpoints and histological variability. The results should therefore be interpreted as hypothesis-generating rather than definitive. In addition, because no directly comparable osteoporotic fracture-healing model treated with MK-4 and MK-7 was available, the administered doses were selected from adjacent preclinical and translational literature and should not be interpreted as osteogenically equivalent.

Finally, bone turnover markers, including CTXI and ucOC, were measured systemically at a single endpoint and may not fully reflect local events at the fracture site. Circulating CTXI and ucOC may be influenced by fracture healing, estrogen-deficiency-related changes in osteocalcin metabolism, assay variability, and pre-analytical handling factors. These limitations should be considered when interpreting the apparent discrepancy between biochemical, histological, and biomechanical findings.

## 6. Conclusions

In this ovariectomized rat femoral fracture model, MK-4 and MK-7 supplementation did not improve functional fracture healing. Although biochemical and histological findings, more pronounced for MK-4, suggested some biological activity, these effects did not translate into mechanically competent union, and both treatment groups showed nonunion-like mechanical behavior at the six-week endpoint. Thus, under the present experimental conditions, vitamin K2 supplementation was insufficient to overcome the impaired healing associated with osteoporotic bone and may not improve and could potentially impair the fracture healing process. No significant hepatic toxicity was observed. Further studies are warranted to clarify whether alternative dosing regimens, treatment timing, or experimental models may better define the role of vitamin K2 in osteoporotic fracture healing.

## Figures and Tables

**Table 1:** Descriptive values of ALT, CTXI, ucOC, BALP and PINP.

	ALT (U/L)	CTXI	ucOC	BALP	PINP
Sham control	79.500 ± 15.000	1799.217 ± 103.005 ^a^	0.208 ± 0.035 ^a^	0.632 (0.172)	2.632 ± 0.482 ^a^
OVX+FR Control	102.250 ± 24.364	1212.650 ± 319.566 ^b^	0.114 ± 0.014 ^b^	0.656 (0.134)	3.459 ± 0.516 ^a^
OVX+FR+MK-4	100.875 ± 26.040	1280.428 ± 172.848 ^b^	0.186 ± 0.045 ^a^	0.760 (0.282)	9.021 ± 3.286 ^b^
OVX+FR+MK-7	113.250 ± 25.138	1121.200 ± 301.310 ^b^	0.182 ± 0.117 ^ab^	0.735 (0.314)	4.580 ± 3.116 ^ab^

Values are presented as mean ± SD for normally distributed variables and median (IQR) for BALP. Study groups, *n* = 8; Sham control group, *n* = 4. For variables with significant global intergroup differences, superscript letters indicate Games–Howell post hoc comparisons. Within the same column, groups sharing at least one superscript letter are not significantly different, whereas groups with no shared superscript letters differ significantly at *p* < 0.05. No superscripts are shown for ALT and BALP because there were no significant intergroup differences.

**Table 2:** Global intergroup comparisons of biochemical endpoints.

	ALT	CTXI	ucOC	BALP	PINP
F or H(df)	1.741	19.859	12.292	H (3) = 2.171	10.152
*p*	0.185	<0.001	0.001	0.538	0.002

Welch’s ANOVA was used when variance homogeneity assumptions were not met (ALT, CTXI, ucOC, PINP). BALP was analyzed using the Kruskal–Wallis test because of non-normal distribution and is reported as H(df).

## Data Availability

Individual animal-level data are provided in Supplementary Table S1. Additional data are available upon reasonable request from the corresponding author.

## Supplementary Materials

The following supporting information can be downloaded at: https://www.mdpi.com/article/10.3390/jcm15124510/s1, Figure S1: Mechanical endpoint values by experimental group; Table S1: Biochemical and mechanical raw data; Table S2: Arrow color legend for histological analysis.
