# Supplementary material for: Discrepancy Between Biological Activity and Functional Fracture Healing Following Vitamin K2 Supplementation in an Ovariectomized Rat Model of Osteoporosis
Source: J Clin Med. 2026 Jun 10;15(12):4510. doi: 10.3390/jcm15124510 (PMC13301873; doi:10.3390/jcm15124510)
Supplement: Supplementary file 1 [file jcm-15-04510-s001.zip › jcm-4307422-supplementary.pdf]

Supplementary Materials:

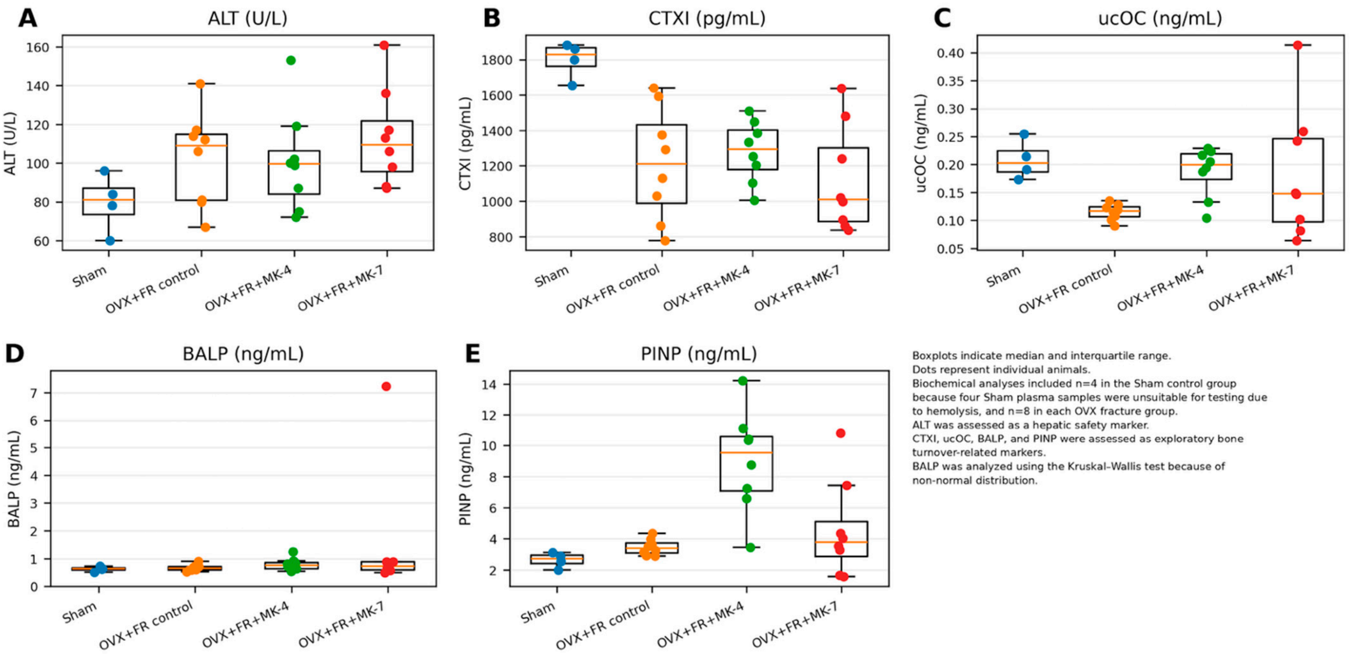

Supplementary Figure S1: Mechanical endpoint values by experimental group.

Supplementary Table S1: Biochemical and mechanical raw data.

No	Group	ALT (U/L)	CTXI (pg/mL)	ucOC (ng/mL)	BALP (ng/mL)	PINP (ng/mL)	Mechanical endpoint (N)	Mechanical Interpretation
1	Sham Control	60,000	1654,450	0,255	0,732	2,000	97,000	Load-to-failure
2	Sham Control	96,000	1883,380	0,173	0,510	3,109	106,000	Load-to-failure
3	Sham Control	NA	NA	NA	NA	NA	NA	Load-to-failure
4	Sham Control	84,000	1860,770	0,191	0,643	2,541	110,000	Load-to-failure
5	Sham Control	NA	NA	NA	NA	NA	NA	Assigned to histological group
6	Sham Control	78,000	1798,270	0,214	0,621	2,881	88,000	Assigned to histological group

7	Sham Control	NA	NA	NA	NA	NA	NA	Assigned to histological group
8	Sham Control	NA	NA	NA	NA	NA	NA	Assigned to histological group
9	OVX+FR Control	114,000	1640,006	0,123	0,533	3,139	38.1	Load-to-failure
10	OVX+FR Control	117,000	1030,489	0,135	0,594	2,909	29.7	Load-to-failure
12	OVX+FR Control	81,000	1130,901	0,108	0,612	4,350	42.2	Load-to-failure
16	OVX+FR Control	141,000	859,586	0,114	0,702	3,981	50.2	Load-to-failure
11	OVX+FR Control	80,000	1376,204	0,090	0,722	3,642	NA	Assigned to histological group
13	OVX+FR Control	106,000	1593,949	0,101	0,582	3,510	NA	Assigned to histological group
14	OVX+FR Control	67,000	1292,398	0,128	0,713	3,251	NA	Assigned to histological group
15	OVX+FR Control	112,000	777,670	0,119	0,900	2,897	NA	Assigned to histological group
17	OVX+FR+MK-4	99,000	1253,728	0,104	0,614	7,234	37,000	Non-interpretable, fail to achieve load to failure, behaving like nonunion-like
18	OVX+FR+MK-4	75,000	1385,506	0,224	0,642	8,750	29,000	Non-interpretable, fail to achieve load to failure, behaving like

								nonunion-like
19	OVX+FR+MK-4	119,000	1004,474	0,133	0,925	10,440	21,000	Non-interpretable, fail to achieve load to failure, behaving like nonunion-like
20	OVX+FR+MK-4	153,000	1510,913	0,187	0,714	11,135	24,000	Non-interpretable, fail to achieve load to failure, behaving like nonunion-like
21	OVX+FR+MK-4	72,000	1447,900	0,229	1,249	10,346	NA	Assigned to histological group
22	OVX+FR+MK-4	100,000	1333,955	0,217	0,806	14,233	NA	Assigned to histological group
23	OVX+FR+MK-4	87,000	1203,282	0,205	0,840	3,457	NA	Assigned to histological group
24	OVX+FR+MK-4	102,000	1103,669	0,194	0,548	6,580	NA	Assigned to histological group
25	OVX+FR+MK-7	88,000	894,419	0,242	0,659	10,835	25,000	Non-interpretable, fail to achieve load to failure, behaving like nonunion-like
26	OVX+FR+MK-7	161,000	1021,038	0,149	0,502	3,543	24,000	Non-interpreta

								ble, fail to achieve load to failure, behaving like nonunion-like
27	OVX+FR+MK-7	98,000	837,105	0,259	0,884	7,424	37,000	Non-interpretable, fail to achieve load to failure, behaving like nonunion-like
28	OVX+FR+MK-7	87,000	996,918	0,414	0,812	4,350	27,000	Non-interpretable, fail to achieve load to failure, behaving like nonunion-like
29	OVX+FR+MK-7	113,000	1637,616	0,147	7,228	3,277	NA	Assigned to histological group
30	OVX+FR+MK-7	136,000	1240,311	0,064	0,893	1,645	NA	Assigned to histological group
31	OVX+FR+MK-7	106,000	1480,974	0,082	0,567	1,574	NA	Assigned to histological group
32	OVX+FR+MK-7	117,000	861,220	0,102	0,605	4,038	NA	Assigned to histological group

Sham control group specimen 3, 5,7,8 have no biochemical data as the plasma samples were not analyzed due to hemolysis.

Supplementary Table S2: Arrow color legend for histological analysis.

Figure	Arrow color	Structure indicated
Figure 1	Black	Fracture diaphyseal ends
Figure 1	Green	Subperiosteal callus
Figure 1	Gray	Hematopoietic marrow
Figure 1	Purple	Periosteal fibrocartilaginous callus
Figure 1	Yellow	Medullary displaced bone fragment
Figure 1	Red	Subperiosteally displaced bone fragment
Figure 1	Pink	Bone fragment located along the fracture line
Figure 2	Black	Fracture diaphyseal ends
Figure 2	Green	Subperiosteal callus
Figure 2	Gray	Hematopoietic marrow
Figure 2	Purple	Periosteal fibrocartilaginous callus
Figure 2	Yellow	(A) Fibrous tissue proliferating around the inserted rod<br>(B) Osseous callus between fracture ends
Figure 2	Red	(A) Subperiosteally displaced fragment<br>(D) Osseous fragment displaced into the medullary cavity
Figure 2	Pink	Bone fragment located along the fracture line
Figure 2	Blue	Fibrous tissue proliferating around the intramedullary rod
Figure 3	Black	Fracture diaphyseal ends
Figure 3	Green	Subperiosteal osseous callus
Figure 3	Gray	Hematopoietic marrow
Figure 3	Purple	Periosteal fibrocartilaginous callus

Figure 3	Blue	(A,B,C) Fibrous tissue proliferating around the rod<br>(D) Fibrocartilaginous callus
Figure 3	Red	Osseous fragment displaced into the medullary cavity
Figure 3	Pink	Osseous callus proliferating from the medullary cavity
Figure 3	Orange	Osseous fragment remaining along the fracture line
